# Prediction of the Effective Work Function of Aspirin and Paracetamol Crystals by Density Functional Theory—A First-Principles Study

**DOI:** 10.1021/acs.cgd.3c00218

**Published:** 2023-07-31

**Authors:** James R. Middleton, Andrew J. Scott, Richard Storey, Mariagrazia Marucci, Mojtaba Ghadiri

**Affiliations:** † School of Chemical and Process Engineering, University of Leeds, Leeds LS2 9JT, United Kingdom; ‡ New Modalities Product Development, Pharmaceutical Technology & Development, Operations, AstraZeneca, Macclesfield SK10 2NA, United Kingdom; § Oral Product Development, Pharmaceutical Technology & Development, Operations, AstraZeneca, Gothenburg 413 27, Sweden

## Abstract

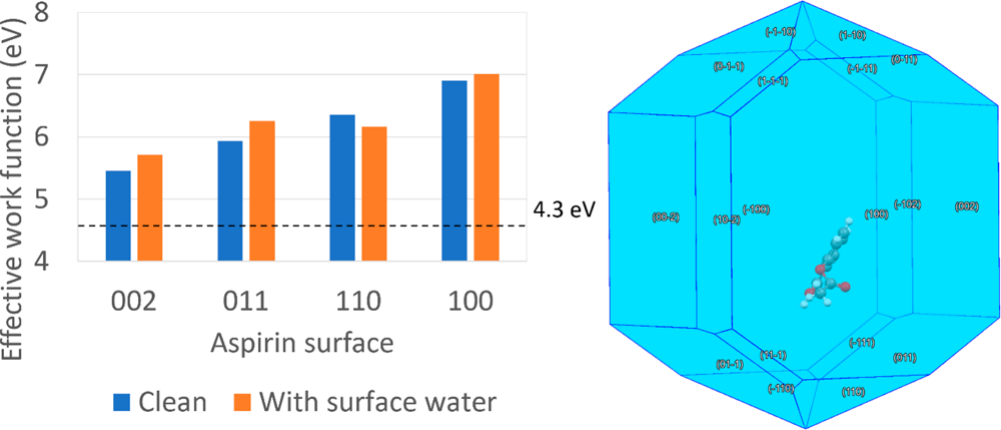

Crystals of active pharmaceutical ingredients (API) are prone to triboelectric charging due to their dielectric nature. This characteristic, coupled with their typically low density and often large aspect ratio, poses significant challenges in the manufacturing process. The pharmaceutical industry frequently encounters issues during the secondary processing of APIs, such as particle adhesion to walls, clump formation, unreliable flow, and the need for careful handling to mitigate the risk of fire and explosions. These challenges are further intensified by the limited availability of powder quantities for testing, particularly in the early stages of drug development. Therefore, it is highly desirable to develop predictive tools that can assess the triboelectric propensity of APIs. In this study, Density Functional Theory calculations are employed to predict the effective work function of different facets of aspirin and paracetamol crystals, both in a vacuum and in the presence of water molecules on their surfaces. The calculations reveal significant variations in the work function across different facets and materials. Moreover, the adsorption of water molecules induces a shift in the work function. These findings underscore the considerable impact of distinct surface terminations and the presence of molecular water on the calculated effective work function of pharmaceuticals. Consequently, this approach offers a valuable predictive tool for determining the triboelectric propensity of APIs.

## Abstract 2

Density Functional Theory calculations are employed to predict the effective work function of different facets of aspirin and paracetamol crystals. The calculations reveal significant variations in the work function across different facets and materials. Moreover, the adsorption of water molecules induces a shift in the work function. These findings underscore the considerable impact of distinct surface terminations and the presence of molecular water on the calculated work function of pharmaceuticals.

## Introduction

Triboelectrification is a widespread phenomenon in which charge transfer occurs between contacting surfaces. This phenomenon commonly arises from sliding or direct impact. In industrial powder processing, such as sieving, fluidizing, conveying, pouring, and grinding, triboelectric charging occurs frequently, leading to substantial electrostatic charge transfer.^1,2^ In a number of applications, electrostatically charged particles have great utility in dry coating,^3^ gas cleaning,^4^ and preventing segregation in some mixtures.^5^ However, unwanted charging can cause severe negative consequences. Particle adhesion to vessel walls, also known as “sheeting”, can cause uneven inlet flow or blockages.^6−10^ Agglomeration and segregation of charged particles can cause blending problems and threaten the homogeneity of powder formulations.^11^ Excessive charge build-up can result in electrostatic discharge, posing a significant fire and explosion risk.^12,13^

The concept of triboelectric charging has been known for thousands of years. The ancient Greeks observed that by rubbing a material against amber it could attract small objects.^14^ Despite this, there is still much that scientists do not fully understand about this phenomenon. Lively debate continues over which mechanism dominates this process, electron transfer or ion transfer.^15^ The magnitude and polarity of charging can vary significantly depending on numerous factors. Mode of contact, i.e. friction, contact or separation,^16^ environmental conditions such as temperature,^17^ relative humidity,^18^ or external electric field,^19^ and material properties such as particle shape and size distribution^20,21^ and surface roughness^22^ all affect charge transfer. Charging tendency varies between different substances, and they are typically ranked into a so-called triboelectric series, which is a list of materials arranged in order of their tendency to become electrically charged when they come into contact with another material.^23^ Pharmaceuticals and excipients have significantly different charging behaviors in both polarity and magnitude.^24^ Interestingly, particles in single-component systems also experience charging when agitated,^20,25^ and it has been shown that contacting nominally identical materials with opposite surface curvature (concave vs convex) will consistently charge positively or negatively depending on curvature.^26^ Also, “flexoelectricity”, the coupling between polarization and strain present in all insulators, is shown to have a measurable effect on triboelectric charge transfer.^27^

The charging of identical materials and the supposed dominance of particle–particle contacts in powder flows^28^ suggest that the cause of triboelectric charging in single-component systems is most likely due to subtle structural differences between surfaces at contact points. The questions which naturally arise are whether the chemistry of the surfaces is anisotropic, does the uneven coating of surface water influence charge transfer, or do the variations in surface electronic structure caused by temperature, mechanical stress, or contamination play a role. Compelling evidence of the importance of water films in contact charging has been presented by Baytekin et al.,^29^ where observed mosaics in surface potential were attributed to surface water. Additionally, Lee et al.^30^ observed that the differences in surface hydrophobicity greatly increase the magnitude of transferred charges. There is a growing amount of research that suggests electron transfer is the dominant process in triboelectrification,^31^ but for adsorbed species on the surface, such as water, ion transfer cannot be ruled out. Thus, further supporting evidence is needed by understanding the dynamics of charge transfer during contact.

Generally, studying the behavior of powder systems is complex as interparticle interactions are influenced by numerous physical and environmental factors, which are practically difficult to “decouple”.^32^ Additionally, electrostatic charging has been described as “unpredictable”,^33^ since triboelectric charging is impacted by both physical properties and environmental conditions. These factors make obtaining reliable experimental results challenging. Many published results are difficult to interpret and in some instances appear contradictory.^15^ Modeling has shown great utility in studying triboelectric charging as computational methods offer precise control of the system in question and can reveal detailed underlying causes. Significant work has already been done on the macroscale modeling of triboelectrification in unit operations. A review of such techniques, applied to fluidized beds, is given by Fotovat et al.^34^ A detailed discussion of current modeling approaches is also given by Chowdhury et al.^35^ However, in both these publications the mechanisms for charging have not been addressed. *A priori* predictions could enable the triboelectric charging to be assessed more effectively.^36^

Density Functional Theory (DFT) is a computational quantum mechanical modeling method that is widely used to study the electronic structure and properties of materials.^37,38^ In recent years it has also been used to model triboelectric charging. Nikitina et al.^39^ applied time-dependent DFT to produce an *ab initio* predicted triboelectric series. It has also been used to study how material deformation and mechanically induced ionization at a surface might impact charge transfer.^40−42^ The work of Lin and Shao^43^ investigated how the presence of surface water can impact the electronic structure of a material, leading to a reversal in the polarity of transferred charges. Shen et al.^44^ simulated charge transfer directly by calculating redistribution of atomic charges between contacting surfaces of quartz and sapphire. Furthermore, a significant amount of research is being devoted to the study of Tribo-Electric Nano-Generators (TENGs)^45,46^ to optimize their design and predict their performance. This has contributed to an increase in the use of DFT to study triboelectrification.^47−52^

There is limited research on applying DFT to study the triboelectric charging of pharmaceutical materials in the literature. However, one notable exception is the work done by Brunsteiner et al.^53^ In this study, the charging behavior of several pharmaceutical materials was predicted from first-principles using DFT by comparing the calculated work function, ionization potential, and the highest occupied molecular orbitals (HOMO) to experimentally obtained charging data.

The work function of a material is an important parameter that can be used to model triboelectric charging. It can be used to predict its tendency to gain or lose electrons during contact and separation.^54^ Photoemission spectroscopy and Kelvin probe force microscopy are common techniques for characterizing the work function of materials and are generally effective at studying metallic and semiconducting materials.^55,56^ However, these methods can struggle to accurately characterize the work function of insulating materials due their intrinsically high resistivity, which causes charge buildup and low electron mobility at the surface, making reliable measurement more difficult.^57,58^ Consequently, research work on the experimental or theoretical determination of the work function for pharmaceutical materials, which are predominantly insulating molecular crystals, is very limited. In recent years, DFT has been used widely to study how crystal orientation,^59,60^ surface chemistry,^61^ deformation,^41^ and surface water^51^ impact work function. It gives good predictions for conducting materials. Its application to insulating crystals shows great promise,^62^ providing a strong methodological basis for the calculation of the work function of pharmaceutical materials, referred to as the “effective” work function (WF).

In this work, DFT is used to predict the effective WF of several crystal facets of paracetamol and aspirin and explore the effect of adsorbed water molecules. Electronic structure calculations are performed using the CASTEP Density Functional Theory package^63^ to determine the electrostatic potential and effective WF and how these quantities change in the presence of water.

## Theoretical Approach

**Figure 1:**
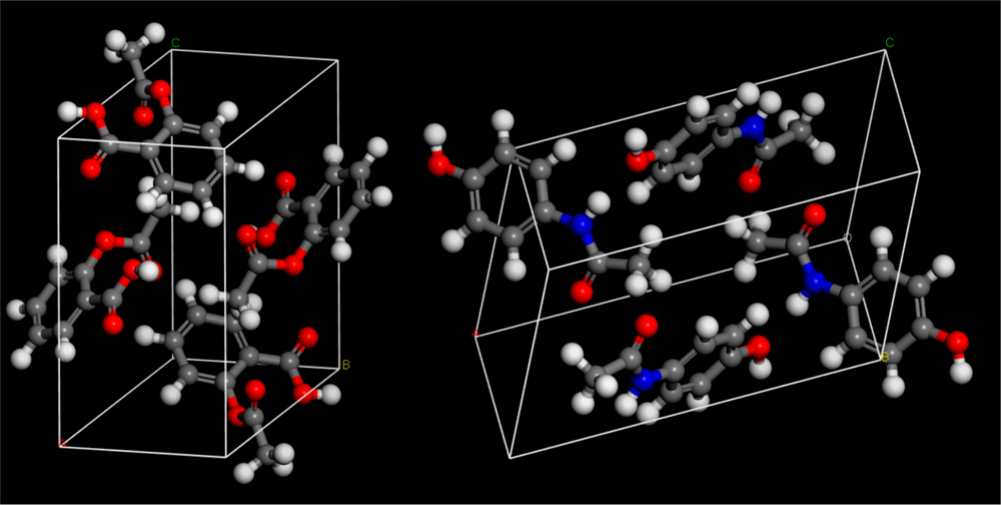
Bulk unit cells of aspirin (ACSALA01) (left) and paracetamol (HXACAN01) (right). Crystallographic Information Files downloaded from the Cambridge Crystallographic Data Centre Web site.^66^

Electronic structure calculations are performed to determine the electrostatic potential and WF within the framework of periodic Density Functional Theory (DFT). The Generalized Gradient Approximation (GGA) Perdew–Burke–Ernzerhof (PBE) exchange-correlation functional is used.^64^ On-The-Fly-Generated (OTFG) ultrasoft pseudopotentials are used in all cases. Dispersion forces are expected in this system, so the Tkatchenko–Scheffler (TS) dispersion correction is applied to account for van der Waals interactions and hydrogen bonding.^65^ The kinetic energy cutoff for the plane wave basis is set at 630 eV to ensure the system is well converged. The crystal structures of aspirin and paracetamol are obtained from the Cambridge Structural Database as a starting point for calculations (Figure 1).^66^ A geometry optimization calculation is then performed on these structures to minimize the total energy of the system with respect to atomic positions. The structures are optimized using the limited memory Broyden–Fletcher–Goldfarb–Shanno (LBFGS) method^67^ until the force acting on each atom is less than 0.01 eV Å^–1^. For geometry optimization calculations of the bulk unit cell, a Monkhorst–Pack grid of (2 × 4 × 2) and (2 × 3 × 4) is used for k-point sampling for aspirin and paracetamol, respectively.

**Figure 2:**
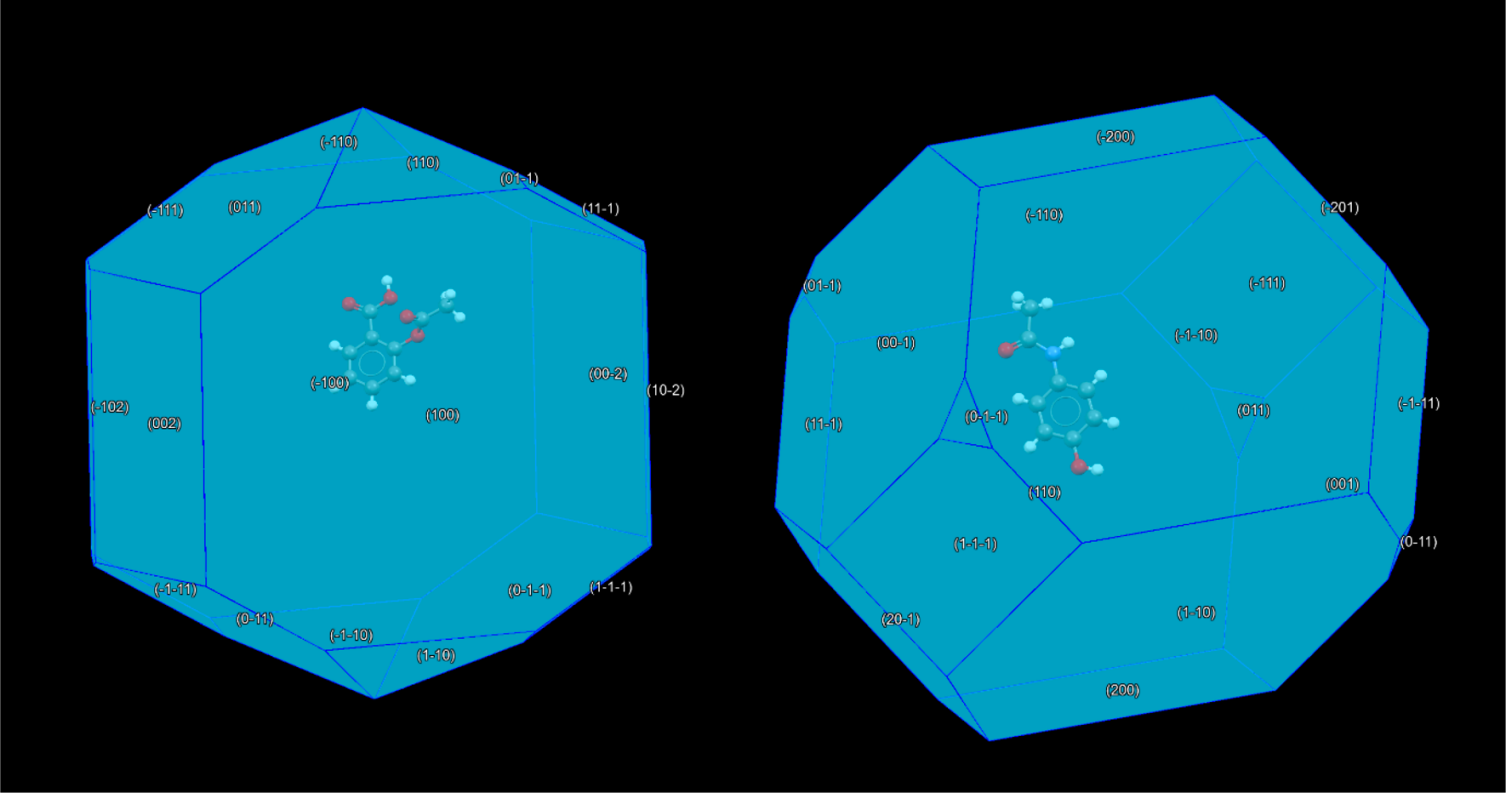
Crystal morphologies of aspirin (left) and paracetamol (right) generated using the BFDH facility in CCDC Mercury.

The shape of these crystals is predicted by using the Bravais, Friedel Donnay, and Harker (BFDH) model. This is a geometrical approach that relates external shape to interplane distance but does not take into account atom type, bonding or partial charges, which all impact crystal growth.^68^ However, this technique has been applied successfully to study the facets of crystalline materials^69−71^ and has also been previously paired with DFT calculations to study surface electronic structure in other studies.^72^ CCDC Mercury software is used to generate the BFDH morphologies of aspirin and paracetamol shown in Figure 2. Four surfaces are selected from each morphology which are considered to represent the primary facets of the crystal and are listed in Table 1.

**Table 1:** Selected Surfaces of Aspirin and Paracetamol with Their Associated Monkhorst–Pack Grid

material	surface (Miller indices).	Monkhorst–Pack grid (*a* × *b* × *c*)
aspirin	(002)	2 × 4 × 1
aspirin	(011)	2 × 2 × 1
aspirin	(110)	2 × 2 × 1
aspirin	(100)	2 × 4 × 1
paracetamol	(200)	3 × 4 × 1
paracetamol	(011)	2 × 3 × 1
paracetamol	(110)	2 × 2 × 1
paracetamol	(001)	4 × 2 × 1

**Figure 3:**
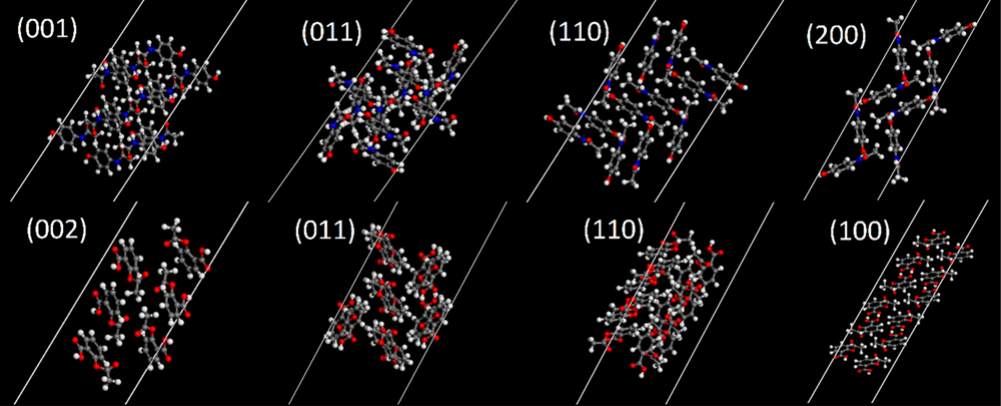
Labeled periodic cells of paracetamol (top) and aspirin (bottom) used in calculations. Visualized using the Materials Studio–Materials Visualizer.

Surfaces are constructed from these optimized structures using the Materials Studio–Materials Visualizer. Supercells are constructed consisting of a thin slab of material separated from its periodic images by a layer of vacuum (Figure 3). To ensure that the top and bottom surfaces of the slab are identical, a slab thickness of *N* unit cells equivalent length is always used. A vacuum gap of 30 Å is used to prevent interaction between periodic slabs so that the vacuum energy can be accurately determined. DFT calculations require that the number of k-points in each direction are inversely proportional to the simulation cell parameters.^73^ The Monkhorst–Pack grid used for each surface is given in Table 1.

**Figure 4:**
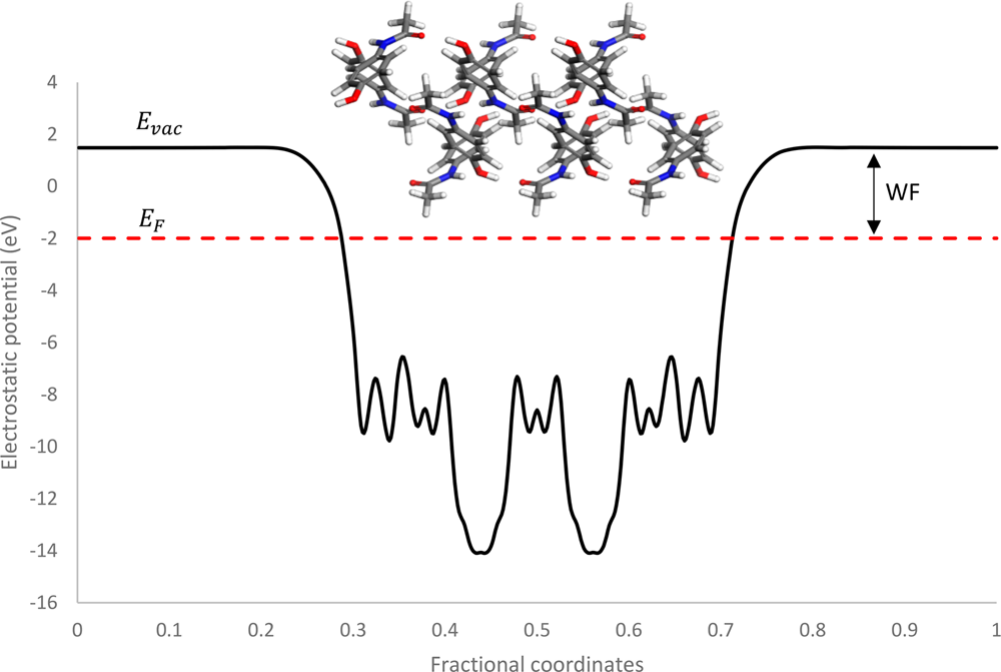
Electrostatic potential of a (001) paracetamol slab. Slab thickness = 23 Å. Vacuum thickness = 30 Å. Fermi energy of the system (*E*_F_), the vacuum energy (*E*_vac_), and the effective WF are labeled in the graph.

**Figure 5:**
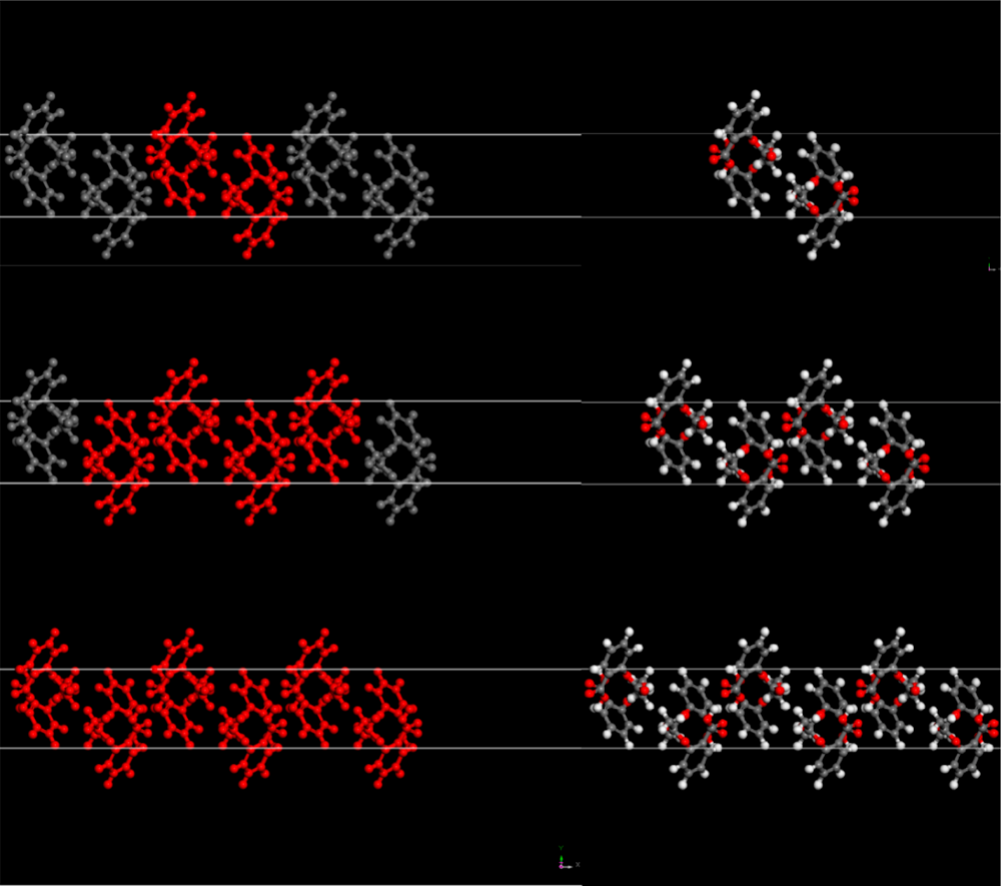
Illustration of different levels of constraint and slab thickness on an aspirin (100) surface: fully constrained (bottom left), one layer unconstrained (middle left), and two layers unconstrained (top left). One unit cell thickness (right top), two unit cells thickness (right middle), and three unit cells thickness (right bottom).

**Figure 6:**
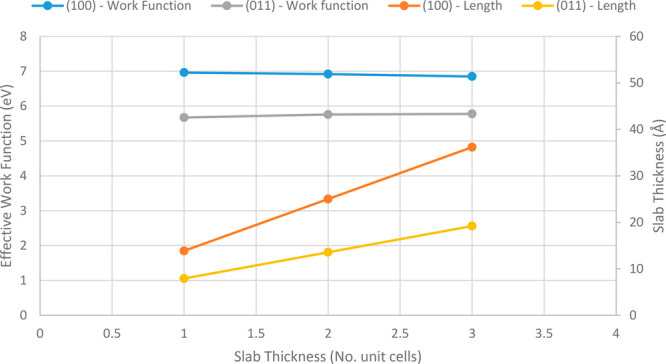
Effect of slab thickness in terms of equivalent unit cell distances on slab length perpendicular to the surface on calculated effective WF.

The work function (WF) is calculated using the equation
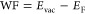
1where *E*_vac_ is the vacuum energy, defined as the electrostatic potential in the vacuum gap when it reaches an asymptotic value. The Fermi energy (*E*_F_), the highest energy electron of the system at 0 K, is calculated at half of the energy gap.^74^ This is shown in Figure 4. A convergence study was carried out testing the dependence of the calculated effective WF on the kinetic energy cutoff, slab thickness, and layers of constrained molecules at the surface. These tests were performed on an aspirin (100) surface (Figure 5), and additional slab-thickness calculations were performed on an aspirin (011) surface for comparison (Figure 6). Testing the dependence of cutoff energy on effective WF, single-point energy calculations were performed on the aspirin (100) surface at values ranging from 25 to 800 eV using a 3*N* unit cell thickness slab. It was found that calculated WF values had converged well by 300 eV. The impact of the number of constrained surface layers was also tested and found to be negligible to the predicted effective WF.

Changes in effective WF due to the number of unconstrained surface layers were found to be negligible. However, one layer of surface molecules was left unconstrained for each surface tested to accommodate structural changes due to the presence of surface water. Figure 6 shows the variation of calculated effective WF with slab thickness, in terms of length and *N* unit cell thickness, for both surfaces tested. The relatively large unit cells associated with aspirin and paracetamol show that a thin slab of one unit cell equivalent length has largely converged. However, a slab thickness equivalent to three unit cells was used for all materials to ensure good electrostatic potential calculations within the bulk and to provide surface layers for structural relaxation in the presence of water.

The surfaces of pharmaceutical molecules are complex and will typically consist of several types of atoms, chemical bonds, and interacting molecules. This creates many local minima, where a water molecule might settle in a geometry optimization calculation. Furthermore, the work of Li et al.^75^ highlights the profound impact that adsorption location can have on the electronic structure of a surface within the context of triboelectric charging. Thus, the role of the aforementioned adsorption location should be considered when optimizing a surface. Due to this, the impact of surface water was also investigated by placing a single molecule of water on each selected surface. A coarse grid search and DFT geometry optimization of the water molecule were performed on each surface to find the lowest energy configuration. The effective WF was then calculated for this structure. The grid search was done using the FORCITE molecular mechanics module of Materials Studio 2021. The Universal force field^76^ was selected to model interactions due to its ready availability and also proven performance in calculating adsorption energies, being in good agreement with experimental results in other studies.^77^

## Results and Discussion

**Figure 7:**
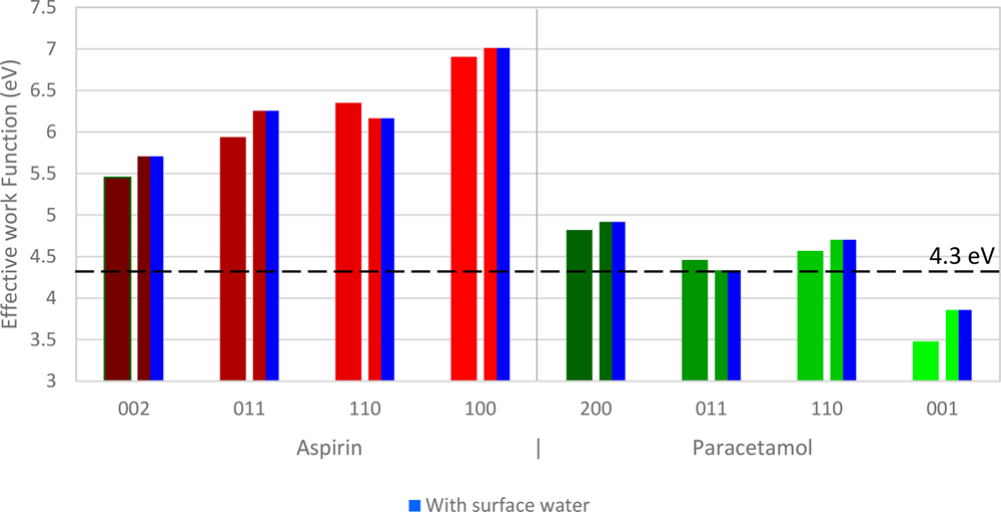
Calculated effective WF of selected surfaces of aspirin (002, 011, 110, 100) and paracetamol (200, 011, 110, 001) and showing the effective WF difference between facets and effective WF shift induced by the fractional coverage of water onto the surface. The dashed line represents the effective WF of stainless steel as reported by Wilson,^82^ and its difference with various facets represents the propensity for charge transfer.

The results of the calculated effective WF of several aspirin and paracetamol surfaces with and without the presence of water are summarized in Table 2. They change significantly depending on both the material and surface tested, as also shown graphically in Figure 7. The highest effective WF observed is associated with aspirin (100) at 6.9 eV with the lowest being paracetamol (001) at 3.5 eV. The range of values observed is itself interesting. A comprehensive review of experimentally determined WF for a wide range of elemental materials has been compiled by Kawano.^78^ The majority of the WFs published in this work are within the range of 2–6 eV. The effective WFs calculated here are in a similar range. Pharmaceutical materials are primarily composed of carbon, hydrogen, oxygen, and nitrogen. However, these calculations show that despite similarities in elemental composition, the differences in the calculated effective WF can be significant. This observation shows the importance of atomic structure, bonding, and surface termination.

**Table 2:** Calculated Effective WF of Selected Surfaces of Aspirin and Paracetamol, Clean and in the Presence of a Single Water Molecule, and Change in Effective WF Due to Water (ΔWF)

		effective work function (eV)		
system	surface	clean	H_2_O	ΔWF (eV)
aspirin	002	5.5	5.7	0.2
aspirin	011	5.9	6.3	0.4
aspirin	110	6.4	6.2	–0.2
aspirin	100	6.9	7.0	0.1
paracetamol	200	4.8	4.9	0.1
paracetamol	011	4.5	4.3	–0.2
paracetamol	110	4.6	4.7	0.1
paracetamol	001	3.5	3.9	0.4

Observing the variation between the surfaces of the same material, the effective WF of both systems varies by up to 1.4 and 1.3 eV for aspirin and paracetamol, respectively. Other works comparing the WF shift due to different surface functionalities are within this range.^61,79^ There is a noticeable distribution of the effective WF between facets. This result is in line with expectations, as anisotropic WFs have been observed in many crystals.^62,78,80^ Even metal surfaces, whose surface terminations are extremely similar, have experimentally verified variations in their WF due to differences in the atomic packing at the surface, which is comparatively minor compared to the differences in surface termination expected for pharmaceutical materials.^81^ It is therefore not surprising that the systems tested in this work also show this effect. Additionally, the effective WF of aspirin is consistently higher than that of paracetamol for all surfaces. The extent of charge transfer between two materials is dependent on the difference in their WF, and a material with a lower WF is expected to transfer electrons to materials with higher WF, resulting in a negative charge for the latter.^54^ The WF of stainless steel is reported as 4.3 eV^82^ and is indicated in Figure 7, which is consistent with the negative polarity and stronger charging propensity of aspirin as compared with paracetamol. The trend is also in line with the experimental work by Šupuk et al.,^24^ who tested the charging propensity of a large number of pharmaceutical powders against stainless steel and found that both aspirin and paracetamol charged negatively against stainless steel, with aspirin charging more strongly. Other calculated effective WF values for pharmaceutically relevant materials are reported in literature; however, this surface anisotropy is typically neglected.^53,83,84^

**Figure 8:**
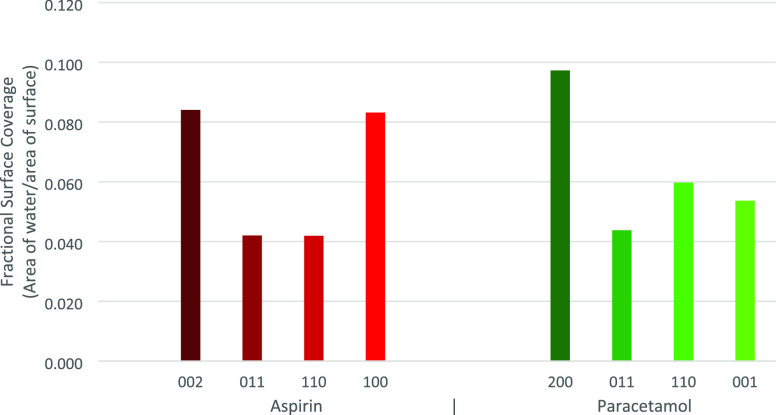
Surface coverage of water for each system, taken as the fractional coverage. The coverage area is based on the circular area of a water molecule with a diameter of 2.8 Å.

**Figure 9:**
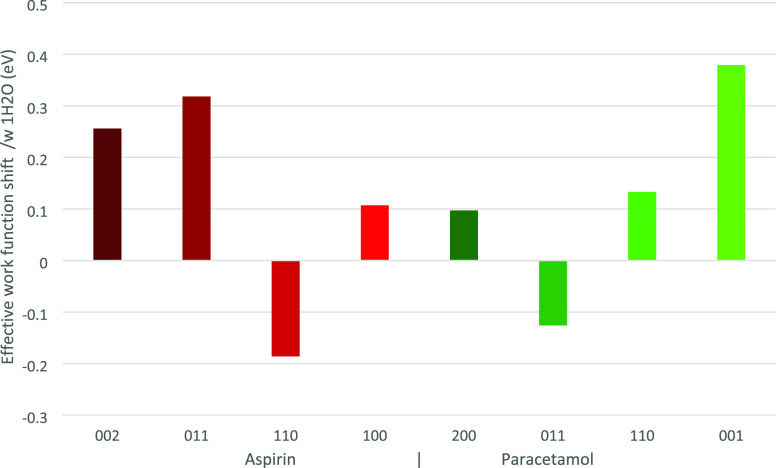
Effective WF shifts due to the presence of water on the surfaces of the selected facet of aspirin (002, 011, 110, 100) and paracetamol (200, 011, 110, 001).

The effect of adsorbed water on the calculated effective WF of each surface was also investigated. The impact of surface chemistry and surface contamination on effective WF are of great interest in other fields.^85,86^ Humidity is known to significantly affect the charging process.^18^ Atomistic studies on surface water in the context of triboelectric charging will typically use either a film-based^51,87^ or molecule-based^75,88^ approach. A film-based approach models multiple layers on the surface, which is arguably more analogous to a real surface; however, it adds significant complexity to the calculation. Molecule-based approaches are less computationally intensive and allow for more detailed study of the different coordinations of water at the surface. In this work, water was simulated on each surface by optimizing a single water molecule onto the surface, similarly to Li et al.^75^ It was found that in the presence of water an effective WF shift in all surfaces was produced (Figure 9). Interestingly, the magnitude of this effective WF shift was found to change depending on which surface the water molecule was placed. The effective WF was found to increase in the presence of water for six out of eight surfaces analyzed, indicating that more energy is required to remove electrons from such surfaces, so a surface with adsorbed water is more likely to get charged negatively. However, for aspirin (110) and paracetamol (011) the effective WF was found to decrease, therefore making it easier for electrons to be removed from the surface. The effective WF shift due to humidity is significant, relevant to the charging of similar materials, which has also been reported in the literature.^25^ This shows that, theoretically, there is an apparent driving force for charge transfer between water-adsorbed and dry surfaces, even for idealized surfaces. This result is also reported by Mukherjee et al.^83^ who calculated a similar decrease in effective WF due to surface water on multicrystalline cellulose. The role of surface coverage was also examined by calculating the fractional coverage of a water molecule on the surface (Figure 8) and comparing it to the magnitude of the WF shift (Figure 9) to determine if it is correlated with the amount of water per unit area. No significant correlation was observed between surface water coverage and effective WF shift. Periodic cell dimensions used in surface coverage calculations are provided in Table 3.

Figure 9 shows an increase and decrease in effective WF depending on the coordination of water molecules on the specific facet, which is very interesting. In the work by Anagaw et al.,^61^ their theoretical calculations suggest that a shift in effective WF is strongly correlated with the surface dipole due to surface modification. In this work, a highly polar molecule, water, is added to the surface. The observation that the effective WF can both increase and decrease in the presence of water shows that the bonding location of the water molecule on the surface is an important consideration.

**Table 3:**
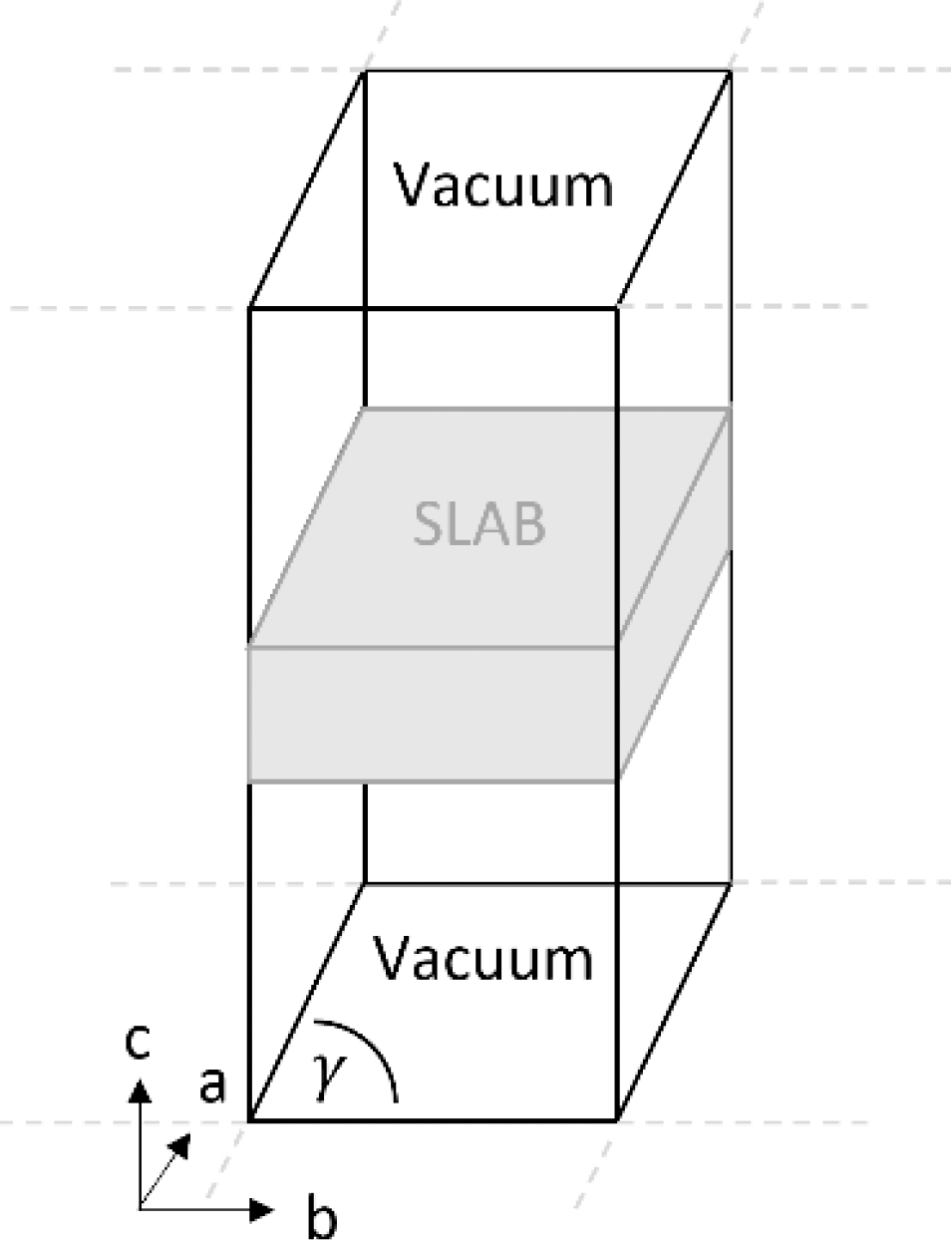
Cross-Sectional Dimensions of Each Unit Cell Axis Normal to the Surface (A and B) and Their Intersecting Angle (γ), with Calculated Values of the Area of Exposed Surface and the Fractional Coverage of Water, Respectively^a^

system	surface	*a* (Å)	*b* (Å)	γ (deg)	exposed surface (Å^2^)	fractional coverage
aspirin	002	11.24	6.51	90.00	73.23	0.08
aspirin	011	11.25	13.10	83.55	146.39	0.04
aspirin	110	11.37	13.00	96.43	146.78	0.04
aspirin	100	6.51	11.37	90.00	73.99	0.08
paracetamol	001	12.68	9.04	90.00	114.66	0.05
paracetamol	011	12.68	11.43	75.73	140.55	0.04
paracetamol	110	7.00	14.84	82.66	103.02	0.06
paracetamol	200	9.04	7.00	90.00	63.28	0.10

a Diameter of water is taken as 2.8 Å based on the work of D’Arrigo.^89^ Illustration of the periodic unit cell is presented above.

**Figure 10:**
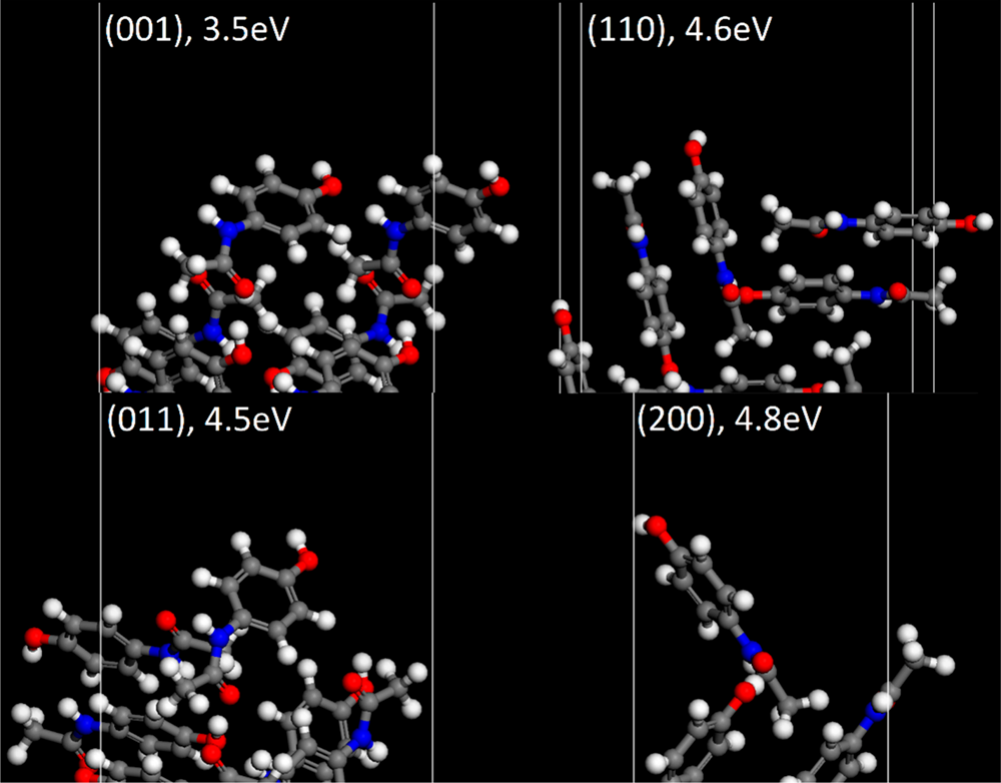
Termination of each paracetamol surface simulated. Calculated effective WF shown above.

**Figure 11:**
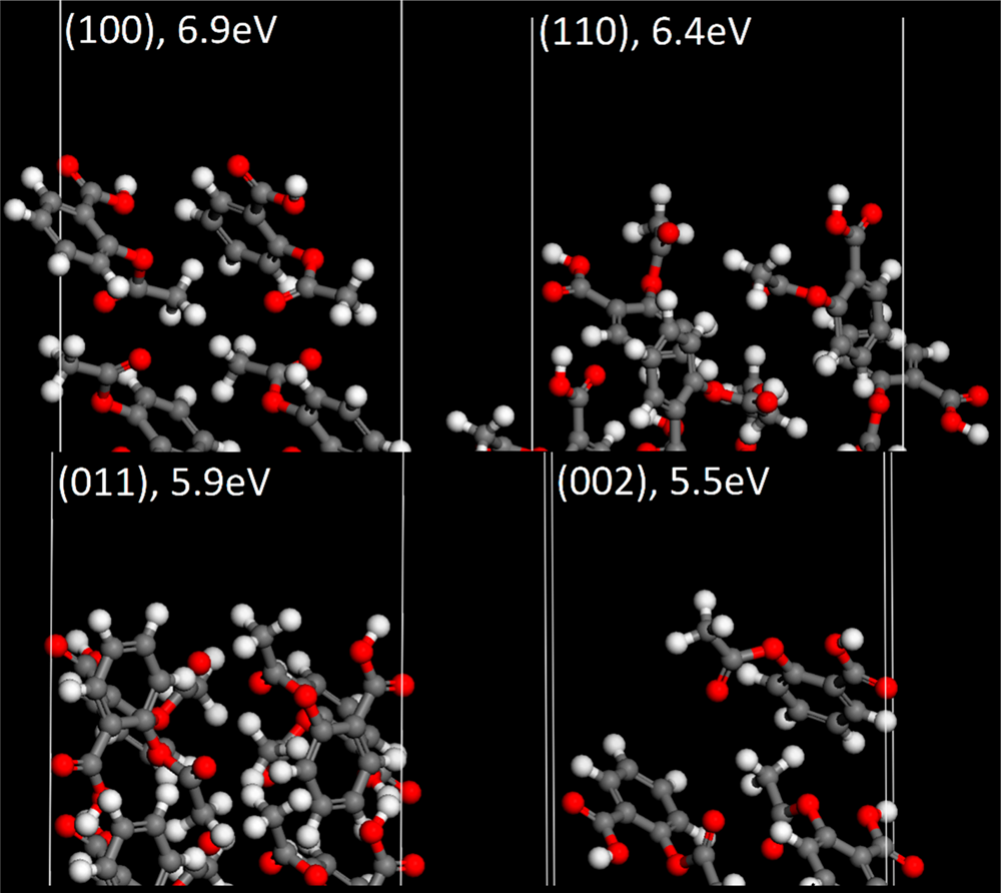
Termination of each aspirin surface simulated. Calculated effective WF shown above.

The surface termination of paracetamol and aspirin is shown in Figures 10 and 11 respectively, with their calculated effective WF values shown. They reveal the different orientations of molecules at the facet and the population of functional groups exposed at the terminating layer. Upon visual inspection of the aspirin surface, there are several electron withdrawing carboxyl groups, placed prominently at the interface. For comparison, Heng et al.^90^ observed that a higher degree of hydrophobicity was observed on the (100) compared with the (011), which is attributed to more prominent carboxyl groups. This is consistent with the surfaces used in this work. Conversely, for paracetamol surfaces there is a greater population of electron donating hydroxyl groups at the surface, and electron withdrawing amide groups are less prominent. In another work, Heng et al.^91^ confirmed the surface anisotropy of paracetamol using X-ray photoelectron spectroscopy, where (001) surfaces were found to have the highest proportion of polar hydroxyl groups, which is also consistent with our model. Anagaw et al.^61^ studied the impact of adsorbed organic molecules on semiconductor surfaces and reported a WF shift due to the electron donating/withdrawing properties of several organic functional groups and the dipole formation at the surface. Therefore, it is possible to hypothesize, in the context of the surfaces of pharmaceutical crystals, that facets with relatively high populations of electron withdrawing groups should be expected to have a high WF, whereas facets with high populations of electron donating groups have a lower WF.

**Figure 12:**
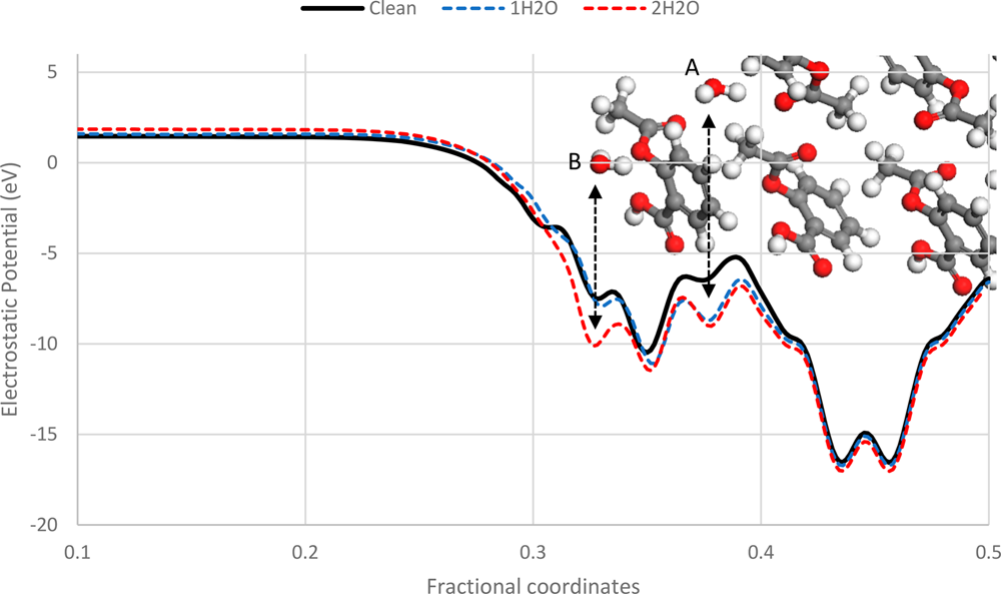
Comparison of the calculated electrostatic potential of aspirin (002) surfaces used to derive the effective WF with and without water molecules. Clean surface (black); single H_2_O molecule adsorbed on the surface of the unit cell, labeled A (blue); two H_2_O molecules adsorbed on surface of the unit cell, labeled A and B (red).

Figure 12 shows the electrostatic potential of the aspirin (002) slab, highlighting a shift in potential caused by the addition of water molecules to the surface. The graph is superimposed onto an image of aspirin (002) as a visual aid to show the position of the molecular water relative to the slab with the water molecules labeled A and B. As previously mentioned, a coarse grid search was performed to determine the most energetically favorable position of the water molecules on each surface. During this search, it was observed that the positions of the water molecules would always converge toward two well-defined locations, depending on the initial search position of the water. Position A is more energetically favorable and taken as the global minimum for this surface, and B is taken as a local minimum. Due to this, aspirin (002) is selected to investigate the impact of multiple water molecules adsorbed onto the surface at different locations.

**Figure 13:**
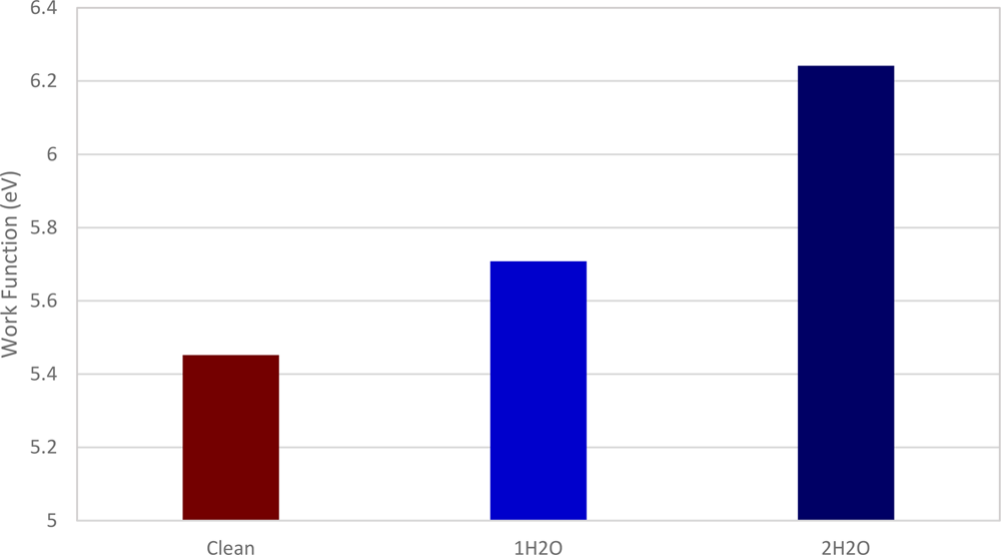
Calculated change in effective WF caused by the addition of water molecules to an aspirin (002) surface.

Comparing the clean surface, shown in black, to the surfaces with adsorbed water, shown in blue and red in Figure 12, shows that the addition of water molecules causes a perturbation in the electrostatic potential toward the surface. The perturbation caused by molecule A is consistent between simulations. The potential energy then quickly adopts bulklike behavior toward the center of the slab (fractional coordinate = 0.5). Since WF is effectively a measure of binding energy of electrons on a surface, the electrons of a material with a higher WF take more energy to remove. For aspirin (002) the addition of several water molecules appears to make this surface more energetically favorable for it to accept electrons (Figure 13).

Our results show that the calculated, effective WF of pharmaceutical materials varies significantly depending on surface and material tested. Similar to other works,^61,85,86,92^ the molecular termination and level of contamination at the surface can be expected to cause a shift in the surface WF. The significance of this relates first to the prediction of charging in pharmaceutical materials, since reliable experimental measurement of these materials can prove difficult to obtain. The work shown here provides a basis on which an understanding of charge transfer between different pharmaceutical crystals can be built. Second, it provides insight into the poorly understood phenomenon of the triboelectrification of chemically identical materials.^93^ Based on this work and the papers previously discussed,^62,78,92^ it is very unlikely that the surface effective WF profile of any particulate solid is homogeneous, implying that triboelectric charge transfer can readily occur for the same material. This has implications for transport and fluidization of homogeneous particulates. These subtle differences in the effective WF and surface electronic structure caused by surface orientation and contamination could provide the driving force for charge transfer in these systems. Finally, the calculated impact of water offers an alternative to the popular belief that the correlation between humidity and triboelectric charging is due to environmental water providing ions for charge transfer. The shift in effective WF caused by adsorbed surface water could itself be facilitating the electron transfer mechanism without necessarily involving ions for the charge transfer.

## Conclusions

The results of this study provide insights into the triboelectric behavior of aspirin and paracetamol crystals by calculating the effective work function of various crystal facets. Significant variations in the effective WF are observed among the facets. Material composition also influences the WF shift. The presence of water molecules on the surface is found to have a noticeable impact, causing changes in the effective WF. This variation may be attributed to the influence of water on the molecular dipole and/or electrostatic potential of the interface, underscoring the importance of atomic coordination and bonding at the surface. Moreover, the calculated effective WF is found to depend on the number of water molecules present, expressed as fractional coverage, highlighting the significance of surface saturation.

This study emphasizes that a substantial distribution of effective WF can be expected in pharmaceutical systems due to surface termination, chemical composition, or surface condition. The findings have implications for understanding charging phenomena in single-component systems and the role of humidity in the charging of pharmaceutical materials. Further research is needed to establish connections between these calculated values and experimental measurements. Currently, there is limited research on facet-specific charging of organic crystals, and expanding the investigations in this area would greatly enhance our understanding of the underlying mechanisms.
